# Supplementary figures and images for: Nonlinear visuoauditory integration in the mouse superior colliculus
Source: PLoS Comput Biol. 2021 Nov 1;17(11):e1009181. doi: 10.1371/journal.pcbi.1009181 (PMC8584769; doi:10.1371/journal.pcbi.1009181)

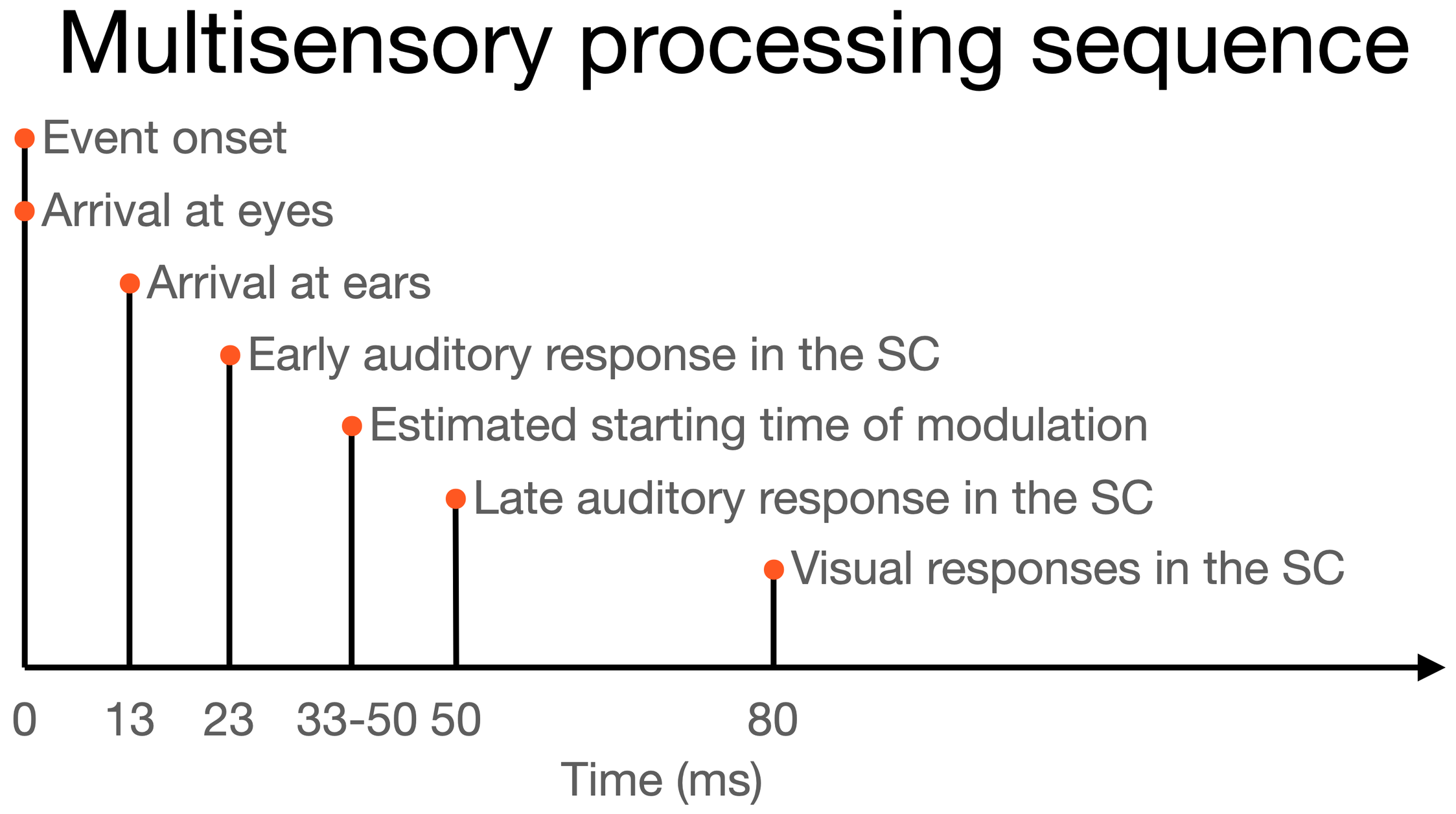

Supplement: S1 Fig — The 13 ms delay in the arrival of the auditory stimulus at the ears is based on a 4.5 m sound source distance and a sound speed of 343 m/s. (TIF) [file pcbi.1009181.s001.tif]

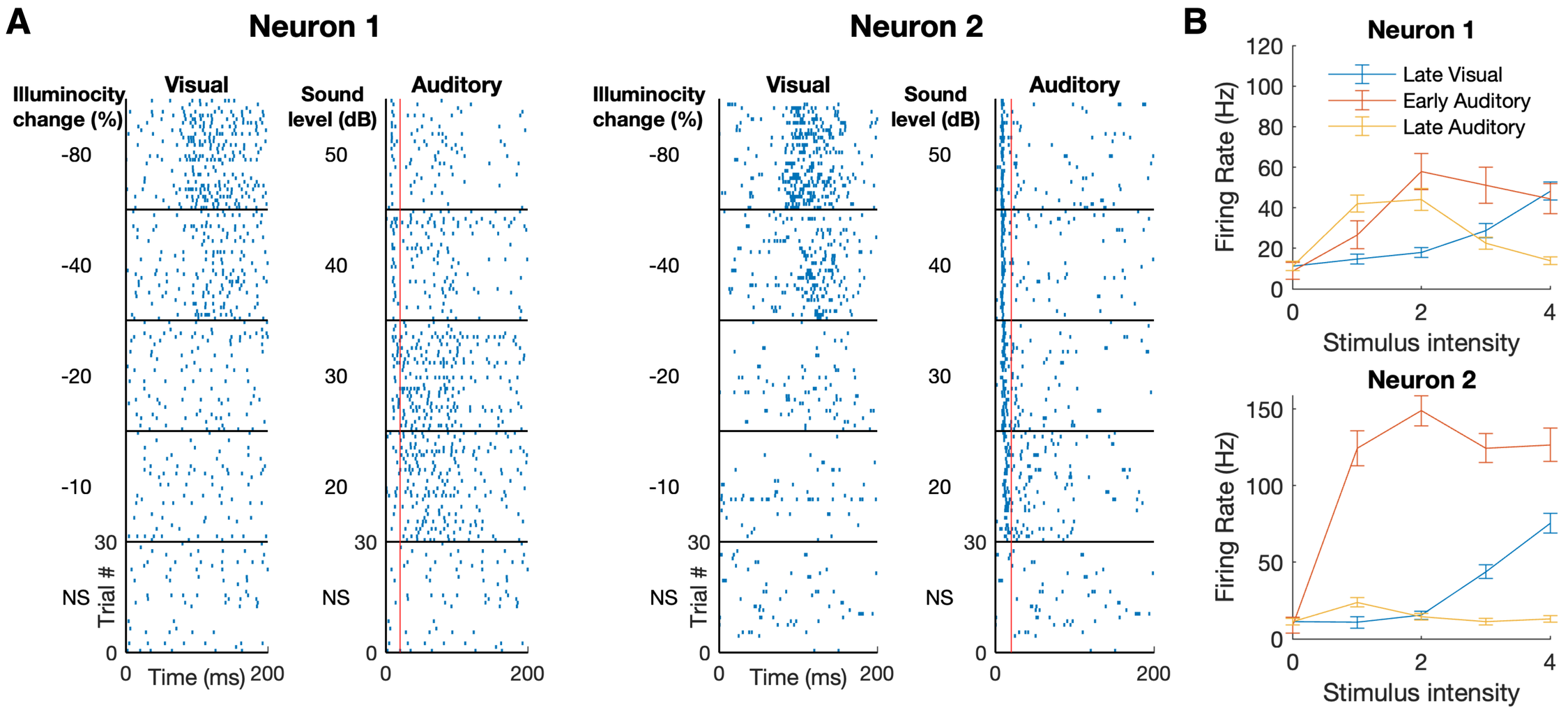

Supplement: S2 Fig — A: Raster plots of two example neurons to different intensities of monomodal visual and auditory stimuli. (NS indicates no stimulus.) These neurons exhibit non-monotonic auditory responses in the late time scale. The red line indicates 20 ms that separates the early and late time windows of auditory responses. B: Summary firing rates of the two neurons in (A). The stimulus intensities are indicated by 0–4 for brevity; their actual values correspond with those used in (A). These neurons’ peak early auditory response is not significantly larger than their max-intensity response (p = 0.25 for neuron 1; p = 0.13 for neuron 2), therefore, these are not considered ‘non-monotonic’ responses. On the other hand, the peak late-auditory response is significantly larger than the max-intensity response (p = 4 x 10^−10^ for neuron 1; p = 0.004 for neuron 2), therefore, these are considered ‘non-monotonic’ responses. (TIF) [file pcbi.1009181.s002.tif]

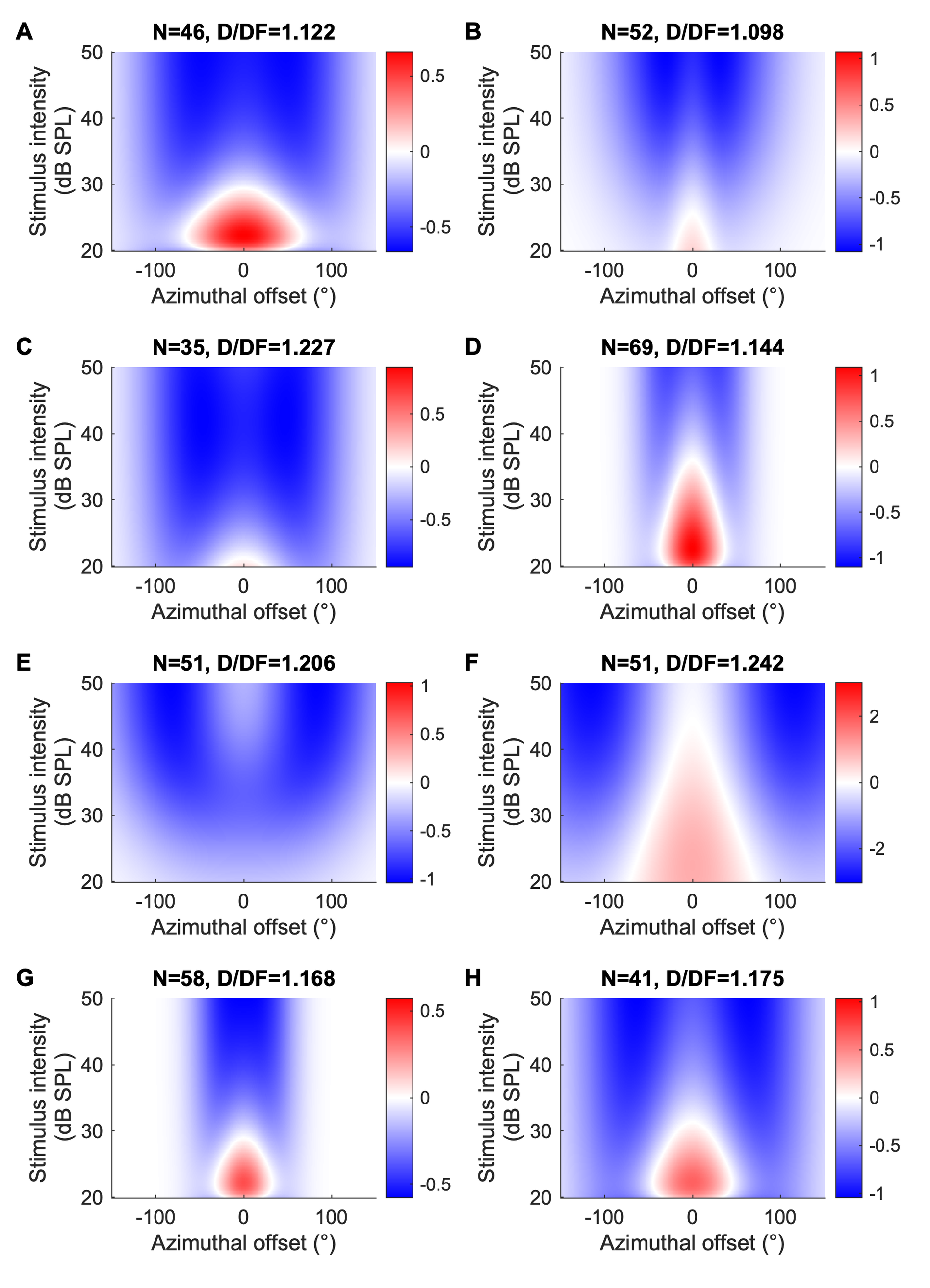

Supplement: S3 Fig — Datasets A-D were used as the exploratory datasets while E-H were used as the blinded datasets (see Materials and methods). N: Number of neurons with late visual or late auditory responses in each dataset. D/DF: Deviance per degree of freedom. (TIF) [file pcbi.1009181.s003.tif]
