# Supplementary material for: Nonlinear visuoauditory integration in the mouse superior colliculus
Source: PLoS Comput Biol. 2021 Nov 1;17(11):e1009181. doi: 10.1371/journal.pcbi.1009181 (PMC8584769; doi:10.1371/journal.pcbi.1009181)
Supplement: Attachment — Submitted filename: Response_to_reviewers.pdf [file pcbi.1009181.s004.pdf]

Response to reviewer comments

We appreciate reviewers' inputs that have led to changes that have improved our manuscript. Below are our point by point answers to the comments of each reviewer. The comments from the reviewers are written in *blue italic font* and our responses are written in black regular font. Changes made in the text are written in red regular font.

Reviewer #1: The authors studied the response properties of mouse SC neurons to spatially restricted visual and auditory stimuli using large-scale physiology. Their data reveal some very important and exciting findings, such as that the mouse SC contains topographically organized visual and auditory neurons; the two visual and auditory maps are aligned; and many neurons exhibit nonlinear multisensory integration. These findings have the potential to form the foundation for future studies on these topics and will be highly interesting to researchers in the field. I have only some suggestions to improve presentation.

1. Unless there is a limit to the number of figures, I suggest that some of the figures be split and some of the supplementary figures moved to main figures to help describe and support the findings.

2. For example, Fig. 1 can be separated into at least 2 figures, with the receptive field data more extensively presented. Quantification of Fig. 1g for all cells that responded to both stimuli, in terms of size, overlap, position correspondence, and depth profile.

We have split Fig. 1 into two figures. In the new manuscript, Fig. 1 presents experimental setup and basic receptive field (RF) measurements. Fig. 2 presents details of the visual and auditory maps. The new Fig. 2 highlights bimodal cells by using a different marker color, and contains new panels that visualize the RF overlaps and depth and azimuthal extent correlations.
The bimodal cells appear only in deep areas of the SC, and we are not aware of any properties of these cells that depend on the details of the depth. These points are addressed in the new text as follows.
The bimodal neurons only appear in dSC where most of the neurons have relatively large visual RFs ($\gtrsim$ 10 °) and their visual/auditory RF azimuthal extent does not correlate with the depth of the cells (Fig. 2E, F). We did not find properties of bimodal neurons that depend on the SC depth.

3. Fig. 2, how is monotonic response defined/quantified? A detailed explanation is needed in the main text.

We added the following explanation that defines monotonicity in the main text.
Here, we call a response 'non-monotonic' when any of the non-maximum strength stimuli elicit a significantly ($p < 0.001$, ANOVA) higher firing rate than the maximum strength stimulus (non-monotonic cells are marked as red in Fig. 3G-I), and otherwise 'monotonic' (these may not satisfy a strict mathematical condition of monotonicity; however, our focus here is to highlight clear non-monotonic responses of a small set of neurons).

4. A major finding of the paper is about non-linear integration between auditory and visual responses, but no examples are shown in the main figure. I suggest moving much of supplementary figure 4 to a main figure, before the model. I understand the statistical issues of conventional approaches to quantify non-linear interactions, but maybe you can use the "best response" to auditory and visual combinations, and then see if that is supralinear or sublinear compared to responses to single stimuli? Better yet, compare this conventional method with the modeling approach to illustrate the model's effectiveness and superior performance.

We separated Fig. 3 in the previous manuscript into two figures. In the new manuscript, Fig. 4 introduces the equation for the model, and Fig. 6 shows the population-level statistics of the model. We inserted Fig. 5, which was previously Supplementary Fig. 4, and with this figure we walk through how the model works for a single neuron. We added the following explanation in the main text to better introduce the model.

Briefly, the model illustrated in Fig. 4 (see Methods) consists of a baseline firing rate (b) plus sigmoidal visual and auditory responses (S), multiplied by a nonlinearity term (modulation coefficient: α^{δ}) that depends on the properties of the auditory stimulus. With this formulation, the nonlinear multisensory interaction is summarized in α^{δ} and its effect on the response (R) can be parameterized from multiple stimulus patterns. The global modulation function δ is defined for each mouse, and applied to all the neurons in the mouse, and in this way, it captures the spatial extent of the modulation in the SC circuitry (see Methods for detail).

Fig. 5 illustrates the example data and the fitting of the model. Fig. 5A-E summarizes the responses of a representative neuron to different levels of visual and auditory stimuli coming from a specific azimuth. The firing rates in the late integration window (Fig. 5C) is compared with the linear additions of the unisensory stimuli (Fig. 5D). For this neuron, the multisensory firing rates are higher than the addition of the unisensory responses when the auditory stimulus is weak (intensities 1 and 2, that correspond to 20 and 30 dB; exhibiting nonlinear multisensory enhancement (MSE)), and lower when the auditory stimulus is strong (intensities 3 and 4, that correspond to 40 and 50 dB; exhibiting nonlinear multisensory suppression (MSS)). The model recapitulates both MSE and MSS, including its dependency on the auditory intensity (Fig. 5G-I).

The neuron in new Fig. 5 contains responses that are representative of the neurons that exhibit multisensory enhancement. We'll use this neuron for describing the nonlinear integration in the data, and how the model works on it.

5. Fig. 3 and the associated model description are a bit too opaque. Maybe use some example cells to illustrate this modeling approach to help readers to understand?

As noted above, the model is now explained together with an example neuron. Then we shift to population-level statistics in new Fig. 6. We hope that this step-by-step introduction would make the model description more transparent.

Reviewer #2: In the current study, the authors examined visual, auditory, and combined responses of SC neurons in mice. The main finding is that there are many cases showing nonlinear visuo-auditory

integration in addition to linear integration. And the authors show that this nonlinear integration factor mainly comes from the auditory modulation, instead from visual. The results are clear. The paper is well written. Here are a few comments and queries.

1. What is the criterion to group “monotonic” and “non-monotonic”?

Our focus was to separate out non-monotonic neurons. We added the following explanation of the definition of monotonicity in the main text.

Here, we call a response ‘non-monotonic’ when any of the non-maximum strength stimuli elicit a significantly ($p < 0.001$, ANOVA) higher firing rate than the maximum strength stimulus (non-monotonic cells are marked as red in Fig. 3G-I), and otherwise ‘monotonic’ (these may not satisfy a strict mathematical condition of monotonicity; however, our focus here is to highlight clear non-monotonic responses of a small set of neurons).

2. The terms of “monotonic” and “non-monotonic” are not proper, because both are monotonic, but just that one is positive and the other is negative as a function of stimulus intensity. Plus, from a few example tuning curves shown, they are not really monotonic, especially for the auditory, by reaching a plateau.

Regarding cells that decrease the firing rate as a function of the intensity, we would agree with the reviewer if the neurons’ spontaneous rate is high and they only decrease the firing rate as the stimulus intensity is increased. However, most of the neurons we labelled non-monotonic have low spontaneous firing rates. Even though the weakest auditory stimulus elicits the maximum firing rate, it is likely that their firing rate decreases if we use weaker stimuli, and smoothly connect to their spontaneous firing rate. Therefore, we do not think our use of the term non-monotonic is invalid in this case.
Regarding neurons that reach a plateau, they can still be called monotonic as they are not decreasing. (A monotonic function that does not permit having the same value is called ‘strictly monotonic’.)
Again, our focus here is rather identifying significant non-monotonic responses (or responses that are tuned to auditory intensity). We hope that saying that explicitly in the text would avoid misleading readers.

3. The authors show that early auditory processing modulates visuoauditory integration, yet through their model, it seems that it can also modulate the visual tuning itself, right? If so, how can visual tuning, especially the monotonic tuning is modulated by auditory early component? Something is puzzling here.

We are not clear how our model suggests the early auditory processing could affect visual tuning, without the context of multisensory integration because the presence of the auditory stimulation does not permit discussing visual tuning alone. If the reviewer is asking the shapes of visual tuning curves with the presence of auditory stimuli with a fixed intensity, we have examples of those shown in new Fig. 5 and Fig. 7C, E. (if you see along the vertical axis of each figure, you get intensity tuning of visual neurons). They are usually modelled well with a sigmoidal tuning curve, and this is consistent with the result in new Fig. 6A that shows the AM-model fits the best to the data.

4. In Fig. 3cd, modulation coefficient refers to δ, whereas in Fig.4b, modulation coefficient refers to α^δ. Suggest to use different terms.

In order to avoid confusion, we called δ 'a global modulation function', α 'a modulation weight', and α^{δ} 'a modulation coefficient' in the new manuscript. In addition, we always used the symbol when these terms appeared in a paragraph for the first time.

5. There appear to be an interaction between the auditory early and late component. However, the authors did not show these data clearly. I would like to see a direction comparison, by a scatter plot, between early and late auditory responses for each neuron. The scatter plot needs to be made for each stimulus intensity, leading to 4-5 plots.

We appreciate the interesting suggestion by the reviewer. We have plotted the requested figure.

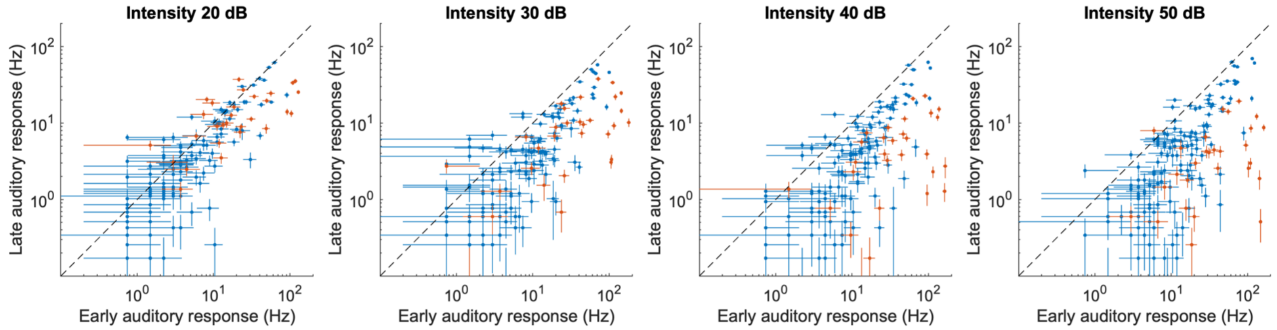

We plotted all of the neurons that exhibit significant responses in both early and late time windows. Neurons indicated by red dots are those that have a non-monotonic response in the late time window. A prominent effect is a general shift towards the right, which indicates that the early auditory response can increase the intensity, while late components do not grow as much as the early component. However, this phenomenon is already communicated in the existing figure (the new Fig. 3 H, I), and this figure may not deliver much new information. Therefore, we have decided not to include this figure in the new manuscript.
